# Levels of Hepatic Activating Transcription Factor 6 and Caspase-3 Are Downregulated in Mice after Excessive Training

**DOI:** 10.3389/fendo.2017.00247

**Published:** 2017-09-26

**Authors:** Ana P. Pinto, Alisson L. da Rocha, Luciana da C. Oliveira, Gustavo P. Morais, Larissa G. de Vicente, Dennys E. Cintra, José R. Pauli, Leandro P. Moura, Eduardo R. Ropelle, Adelino S. R. da Silva

**Affiliations:** ^1^Postgraduate Program in Rehabilitation and Functional Performance, Ribeirão Preto Medical School, University of São Paulo (USP), Ribeirão Preto, Brazil; ^2^School of Physical Education and Sport of Ribeirão Preto, University of São Paulo (USP), Ribeirão Preto, Brazil; ^3^Sport Sciences Course, Faculty of Applied Sciences, State University of Campinas (UNICAMP), Limeira, Brazil

**Keywords:** overtraining, endoplasmic reticulum stress, apoptosis, heart, liver

## Abstract

Recently, we demonstrated that different running overtraining (OT) protocols with the same external load, but performed downhill (OTR/down), uphill (OTR/up), and without inclination (OTR), led to hepatic fat accumulation. As the disruption of endoplasmic reticulum (ER) homeostasis is linked to animal models of fatty liver disease, we investigated the effects of these OT models on the proteins related to ER stress (i.e., BiP, inositol-requiring enzyme 1, protein kinase RNA-like endoplasmic reticulum kinase, eIF2alpha, ATF6beta, and glucose-regulated protein 94) and apoptosis (C/EBP-homologous protein, Caspase-3, 4, and 12, Bax, and tumor necrosis factor receptor-associated factor 2) in livers of C57BL/6 mice. Also, aerobic training can attenuate cardiac ER stress and improve exercise capacity. Therefore, we investigated whether the decrease in performance induced by our OT protocols is linked to ER stress and apoptosis in mouse hearts. The rodents were divided into six groups: naïve (N, sedentary mice), control (CT, sedentary mice submitted to the performance evaluations), trained (TR), OTR/down, OTR/up, and OTR groups. Rotarod, incremental load, exhaustive, and grip force tests were used to evaluate performance. After the grip force test, the livers and cardiac muscles (i.e., left ventricle) were removed and used for immunoblotting. All of the OT protocols led to similar responses in the performance parameters and displayed significantly lower hepatic ATF6beta values compared to the N group. The OTR/down group exhibited lower liver cleaved caspase-3 values compared to the CT group. However, the other proteins related to ER stress and apoptosis were not modulated. Also, the cardiac proteins related to ER stress and apoptosis were not modulated in the experimental groups. In conclusion, the OT protocols decreased the levels of hepatic ATF6beta, and the OTR/down group decreased the levels of hepatic cleaved caspase-3. Also, the decrease in performance induced by our OT models is not associated with ER stress and apoptosis in mice hearts.

## Introduction

The endoplasmic reticulum (ER) is a dynamic organelle of the eukaryotic cells with a central role in protein and lipid biosynthesis (1, 2), in which polypeptides are synthesized from messenger RNA (mRNA). When the ER suffers physiological disturbances that increase the synthesis of unfolded and misfolded proteins, an adaptive response, also known as unfolded protein response (UPR), is triggered (2, 3). Reticular function monitoring and UPR signaling are controlled by three proteins associated with the ER membrane: the inositol-requiring enzyme 1 (IRE1), protein kinase RNA-like endoplasmic reticulum kinase (PERK), and activating transcription factor 6 (ATF6). These proteins remain inactive due to their connections with a binding protein (BiP) chaperone in the intraluminal domains. However, in response to stress situations such as an excess of immature proteins in the ER, the chaperones are recruited, reducing their associations with IRE1, PERK, and ATF6 (2). The glucose-regulated protein 94 (GRP94) is another ER-resident chaperone, which plays a major role in protein folding and ER quality control (4).

When activated, IRE1 oligomerizes and leads to the trans-autophosphorylation of its cytosolic domain and RNase activity. Increased RNase activity cleaves the mRNA of Xbox-binding proteins (XBP1) in the cytosol, which generates spliced XBP1 mRNA encoding a basic leucine zipper (bZIP)-containing transcription factor that increases the ER-associated degradation (ERAD) components and ER chaperones (5). Also, IRE1 can bind to the tumor necrosis factor receptor-associated factor 2 (TRAF2), which activates the apoptosis signal-regulating kinase 1 (ASK1) and forms the IRE1–TRAF2–ASK1 ternary complex. This compound may activate the nuclear factor kB (NF-kB) that exerts anti or proapoptotic effects depending on the cell type and physiological context. IRE1 can also interact with components of the cell-death machinery such as caspase-12 and activates the stress-induced c-Jun N-terminal kinase (JNK), which phosphorylates and inactivates the antiapoptotic regulator BCL-2, leading to Bax-dependent apoptosis (1, 2).

Protein kinase RNA-like endoplasmic reticulum kinase induces phosphorylation of the alpha subunit of eukaryotic translation initiation factor-2 (eIF2alpha) at serine 51 (6). Chronic activation of PERK increases the expression of the C/EBP-homologous protein (CHOP), also known as GADD153, by the activating transcription factor 4 (ATF4). Increased CHOP levels lead to apoptosis by Bax translocation from the cytosol to mitochondria and lower activation of BCL-2 expression (2, 7). ATF6 is translocated from the ER to the Golgi complex and is first cleaved by site 1 protease and then by site 2 protease in the intramembrane region, which leads to the release of the cytosolic DNA-binding portion, ATF6f (“f” for fragment). ATF6f moves to the nucleus and activates a subset of UPR target genes (8). According to Yoshida and coworkers (9), ATF6 is also related to the transcriptional activation of XBP1.

The mechanisms above aim to protect against cell death as well as to decrease overall synthesis and increase chaperone production. However, when UPR persists, the synthesis of immature proteins is not reduced, which results in apoptosis that probably involves other mediators such as caspases 4 and 12, JNK, and CHOP (1). As a non-pharmacological strategy, several studies have investigated the effects of physical exercise on ER stress in different conditions (10–15). Bourdier and coworkers (16) verified that Wistar rats submitted to high-intensity training did not present significant alterations of the pPERK, peIF2alpha, ATF4, ATF6, XBP1, CHOP, and caspase-3-cleaved proteins in the myocardium. However, obese animals submitted to a swimming training protocol reduced the pro-inflammatory molecules (JNK, NF-kB) and ER stress proteins (PERK and eIF2alpha) in adipose and hepatic tissues (10).

Kim et al. (17) verified UPR activation in human skeletal muscle after running 200 km and highlighted that the therapeutic effects of exercise are strictly dependent on its intensity. Based on the cross talk between ER stress and inflammation (18, 19), our research group (20) tested the responses of some ER stress proteins in skeletal muscle samples of mice classified in the non-functional overreaching (NFOR) state. According to Meeusen and coworkers (21), the NFOR state results from an intensified period of training, also known as overtraining (OT), and is characterized as a decline in performance that may be reversed after weeks or months of recovery and may be related to psychological and hormonal alterations.

Regarding NFOR etiology, Smith (22, 23) proposed the cytokine hypothesis speculating that an imbalance between high-load training sessions and sufficient recovery periods leads to musculoskeletal trauma, increasing the release of interleukin 1beta (IL-1beta), IL-6, and tumor necrosis factor alpha (TNF-alpha) (22, 23). High serum levels of pro-inflammatory cytokines would communicate with other organic systems, triggering most of the signs and symptoms previously related to NFOR. Our research group verified that different running OT protocols with the same external load (i.e., intensity versus volume), but performed downhill (OTR/down), uphill (OTR/up), and without any inclination (OTR), increased the serum levels of IL-1beta, IL-6, and TNF-alpha (24), which corroborates with the cytokine hypothesis.

Recently, using immunohistochemistry staining, da Rocha et al. (25) observed hepatic upregulation of IL-6 after the OTR/down and OTR/up protocols, and upregulation of TNF-alpha after the OTR/up protocol. Also, the three OT models led to hepatic fat accumulation (25). As it is known that the disruption of ER homeostasis is linked to animal models of fatty liver disease (26, 27), the aim of this study was to investigate the effects of the OTR/down, OTR/up, and OTR protocols on the proteins related to ER stress (i.e., BiP, IRE1, PERK, eIF2alpha, ATF6beta, and GRP94) and apoptosis (CHOP, Caspase-3, 4, and 12, Bax, and TRAF2) in livers of C57BL/6 mice. Because aerobic training can attenuate cardiac ER stress and improve exercise capacity (14), we also investigated whether the decrease in performance induced by our OT models is linked to ER stress and apoptosis in hearts of C57BL/6 mice.

## Materials and Methods

### Experimental Animals

Eight-week-old male C57BL/6 mice from the Central Animal Facility of the Ribeirão Preto campus were kept in individual cages at a controlled temperature (22 ± 2°C) on a 12:12-h light–dark inversion cycle with food (Purina chow) and water *ad libitum*. The experimental procedures were performed according to the Brazilian College of Animal Experimentation (COBEA) and were approved by the Ethics Committee of the University of Sao Paulo (ID 14.1.873.53.0). The rodents were randomly divided into naïve (N; sedentary mice; *n* = 10), control (CT; sedentary mice submitted to the performance evaluations; *n* = 10), trained (TR; *n* = 10), overtrained by downhill running (OTR/down; *n* = 10), overtrained by uphill running (OTR/up; *n* = 10), and overtrained by running without inclination (OTR; *n* = 10). The mice were manipulated, trained, and overtrained in a dark room between 6:00 a.m. and 8:00 a.m.

### Incremental Load Test (ILT)

First, the mice were adapted to treadmill running (INSIGHT^®^, Ribeirão Preto, São Paulo, Brazil) for 5 days, for 10 min/day at 3 m/min. The initial intensity of the ILT was 6 m/min at 0% with increasing increments of 3 m/min every 3 min until exhaustion, defined when the rodents touched the treadmill end five times in 1 min. The animals were encouraged by physical prodding, and when they became exhausted without finishing the stage, the exhaustion velocity was corrected as proposed by Kuipers and coworkers (28). The EV of each rodent was used to prescribe the intensities of the training or OT protocols (29–31). The speed (m min^−1^) of the ILT for the experimental groups during the evaluation weeks were (1) week 0: CT = 25.5 ± 2.8, TR = 22.0 ± 3.5, OTR/down = 20.7 ± 2.8, OTR/up = 24.2 ± 2.7, and OTR = 23.7 ± 2.5; (2) week 4: CT = 24.3 ± 2.5, TR = 27.7 ± 3.4, OTR/down = 27.3 ± 3.4, OTR/up = 27.3 ± 1.6, and OTR = 29.7 ± 0.9; (3) week 8: CT = 19.8 ± 3.3, TR = 31.0 ± 6.3, OTR/down = 15.3 ± 3.1, OTR/up = 17.1 ± 2.3, and OTR = 18.3 ± 3.4.

### Training Protocol

The 8-week training protocol was based on the study from Ferreira et al. (32) and consisted of 5 days of continuous training interposed by 2 days of recovery (Table 1).

**Table 1:** Characteristics of the training (TR) protocol.

Experimental weeks	Intensity (% EV)	Volume (min)	Frequency	Recovery (h)	Treadmill grade (%)
1	60	15	1/day	24	0
2	60	30	1/day	24	0
3	60	45	1/day	24	0
4	60	60	1/day	24	0
5–8	60	60	1/day	24	0

### Running OT Protocols and Performance Evaluations

The 8-week running OT protocols were performed as previously described (20, 24, 25, 33) and also consisted of 5 days of continuous training interposed by 2 days of recovery (Table 2). The performance evaluations were applied on week 0 and 48 h after the last sessions of the TR and OT protocols at the end of weeks 4 and 8 and consisted of the rotarod test, the ILT, the exhaustive test, and the grip force test (24, 33–36). A detailed description of these performance evaluations has been previously published in other studies by our research group (24, 33, 36, 37). For the performance tests, we used 10 animals from each group. Interestingly, regardless of the predominance of the muscle contraction type used during the OT models, the training volume performed by each group during the experimental weeks was similar (33, 37).

**Table 2:** Characteristics of the running overtraining (OT) protocols performed in downhill, uphill and without inclination.

Experimental weeks	Intensity (% EV)	Volume (min)	Frequency	Recovery (h)	Treadmill grade (%)		
					OTR/down	OTR/up	OTR
1	60	15	1/day	24	0	0	0
2	60	30	1/day	24	0	0	0
3	60	45	1/day	24	0	0	0
4	60	60	1/day	24	0	0	0
5	60	60	1/day	24	−14	14	0
6	70	60	1/day	24	−14	14	0
7	75	75	1/day	24	−14	14	0
8	75	75	2/day	4	−14	14	0

*OTR/down, overtrained by downhill running; OTR/up, overtrained by uphill running; OTR, overtrained by running without inclination*.

### Heart and Liver Extractions

The rodents were anesthetized 36 h after the grip force test. After an overnight fast (12 h), the mice were anesthetized using an intraperitoneal injection of 2-2-2 tribromoethanol 2.5% (10–20 µL g^−1^). Once anesthesia was confirmed by the loss of pedal reflexes, each mouse liver and cardiac muscle (i.e., left ventricle) were removed and used for immunoblotting. For the immunoblotting technique, we used 6 or 8 animals from each group for each protein.

### Immunoblotting Technique

Livers and cardiac muscles (i.e., left ventricles) were homogenized in extraction buffer (1% Triton X-100, 100 mM Tris, pH 7.4, containing 100 mM sodium pyrophosphate, 100 mM sodium fluoride, 10 mM EDTA, 10 mM sodium vanadate, 2 mM PMSF, and 0.1 mg/ml aprotinin) at 4°C with a Polytron PTA 20S generator (model PT 10/35; Brinkmann Instruments, Westbury, NY, USA), operated at maximum speed for 30 s. The homogenates were centrifuged (9,900 *g*) for 40 min, and the supernatants were used for protein quantification using the Bradford method (38). Proteins were denatured by boiling in Laemmli sample buffer containing 100 mM DTT, run on SDS-PAGE gel, and transferred onto nitrocellulose membranes (GE Healthcare, Hybond ECL, RPN303D). The transfer efficiency onto nitrocellulose membranes was confirmed by brief staining of the blots with Ponceau red stain. These membranes were blocked with Tris-buffered saline (TBS) containing 5% BSA and 0.1% Tween-20 for 50 min at room temperature.

The antibodies used for immunoblotting overnight at 4°C were Bax (CELL2772s), beta-actin (CELL4967s), Caspase-4 (CELL4050s), Caspase-12 (CELL2202s), CHOP (CELL2895s), TRAF2 (CELL4712s), phospho-TRAF2 (Ser11; CELL13908s) from Cell Signaling Technology (Beverly, MA, USA) at a dilution of 1:1,000; beta-actin (SC69879), BiP (SC33757), Caspase-3 (SC7148), eIF2alpha (SC11386), phospho-eIF2alpha (Ser52; SC101670), GRP94 (SC11402), PERK (SC13037), and phospho-PERK (Thr981; SC32577) from Santa Cruz Biotechnology (Santa Cruz, CA, USA) at a dilution of 1:750; IRE1 (A37073) and phospho-IRE1 (Ser724; AB104157) from Abcam (Cambridge, UK) at a dilution of 1:1,000; ATF6beta (orb155755), and phospho-Bax (Ser184; orb4658) from Biorbyt (Cambridge, UK) at a dilution of 1:1,000.

After the membranes had been washed with TBS containing 0.1% Tween-20, they were incubated for 1 h at 4°C with secondary antibody conjugated with horseradish peroxidase. The specific immunoreactive bands were detected using chemiluminescence (GE Healthcare, ECL Plus Western Blotting Detection System, RPN2132). Images were acquired by the C-DiGit™ Blot Scanner (LI-COR^®^, Lincoln, NE, USA) and quantified using the Image Studio software for C-DiGit Blot Scanner.

### Statistical Analysis

The results are expressed as the mean ± SEM. While the Shapiro–Wilk *W*-test was used to test data normality, the Levene test was used to test the homogeneity of variances. When normality was confirmed, the one-way ANOVA was used to examine the effects of the TR and OT protocols. When one-way ANOVA indicated statistical significance, a Bonferroni *post hoc* test was performed. When normality was not confirmed, the Kruskal–Wallis test was used to examine the effects of the TR and OT protocols. When Kruskal–Wallis indicated statistical significance, a Games–Howell *post hoc* test was performed. All the statistical analyses were two-sided, and the significance level was set at *P* < 0.05. The statistical analyses were performed using the *SPSS* v.20.0 for Windows software (IBM, Chicago, IL, USA).

## Results

### Performance Parameters

**Figure 1:**
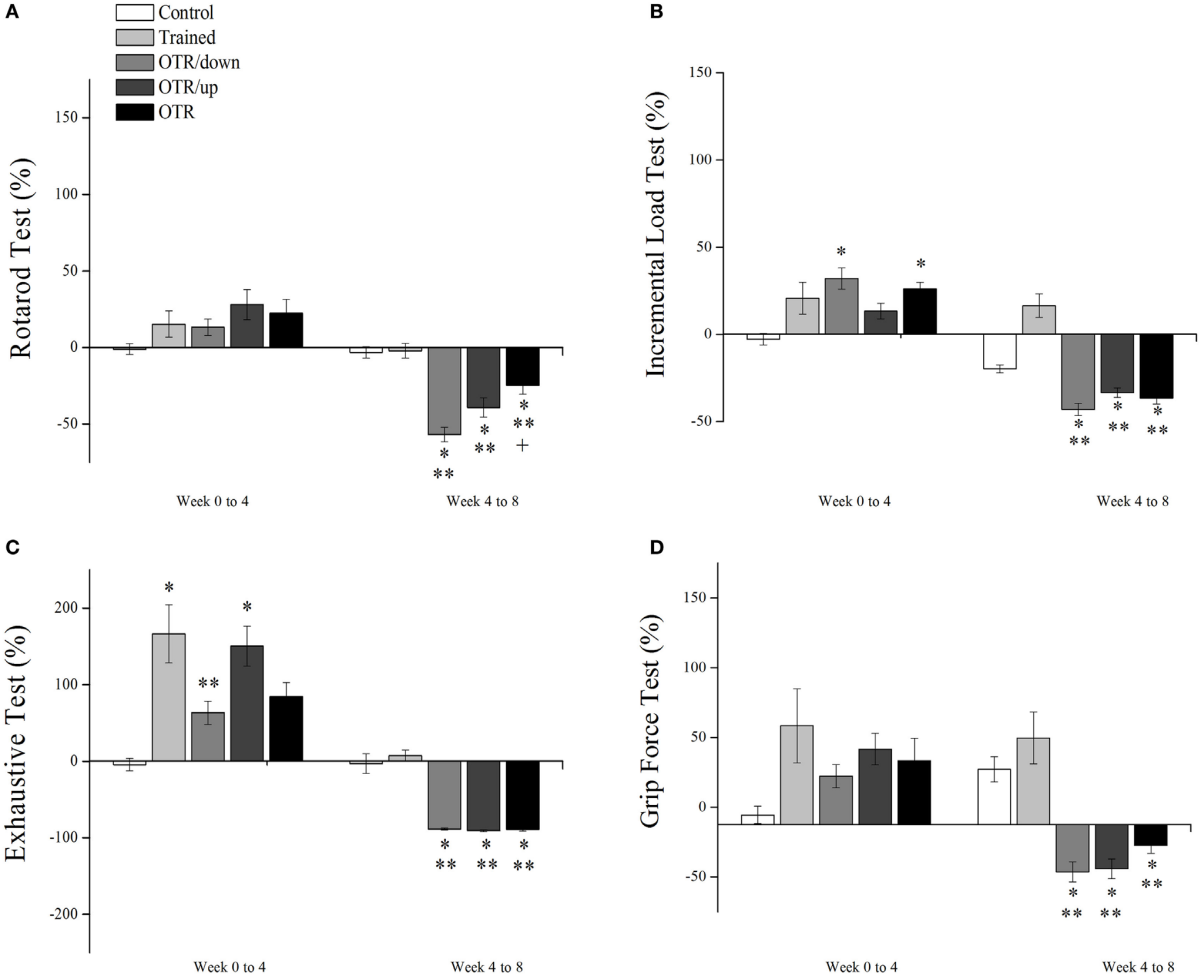
Percentage alterations (%) in rotarod test **(A)**, incremental load test **(B)**, exhaustive test **(C)**, and grip force test **(D)** from week 0 to week 4, and from week 4 to week 8. Data are expressed as the mean ± SE of *n* = 10 mice. CT, sedentary mice; TR, trained mice; OTR/down, overtrained by downhill running; OTR/up, overtrained by uphill running; OTR, overtrained by running without inclination. *P < 0.05 vs. CT group. **P < 0.05 vs. TR group. ^†^*P* < 0.05 vs. OTR/down group.

Figure 1A shows that the alteration (%) in the rotarod test performance from week 0 to week 4 was not significantly different between the experimental groups. From week 4 to week 8, all OT groups decreased their performance significantly compared to the CT and TR groups. Also, the OTR group was significantly lower compared to the OTR/down group. Figure 1B shows that the alteration (%) in the ILT performance from week 0 to week 4 was significantly higher for the OTR/down and OTR groups compared to the CT group.

From week 4 to week 8, the TR group increased its ILT performance significantly compared to the OTR/down, OTR/up, and OTR groups. On the other hand, the OTR/down and OTR groups decreased their ILT performances significantly compared to the CT group. As it is known that a decrease or stagnation in performance is the only marker for the diagnosis of the NFOR state (21, 39–42), our performance results (i.e., Figures 1A–D from week 4 to week 8) reinforce our having established three successful models of OT for mice.

Figure 1C shows that the alteration (%) in the exhaustive test performance from week 0 to week 4 for the TR and OTR/up groups was significantly higher compared to the CT group. Also, the TR group increased its performance significantly compared to the OTR/down group. From week 4 to week 8, all OT groups decreased their performances significantly compared to the CT and TR groups. Figure 1D shows that the alteration (%) in the grip force performance (%) from week 0 to week 4 was not significantly different between the experimental groups. From week 4 to week 8, all OT groups decreased their performances significantly compared to the CT and TR groups.

### Proteins Related to ER Stress and Apoptosis in the Liver

**Figure 2:**
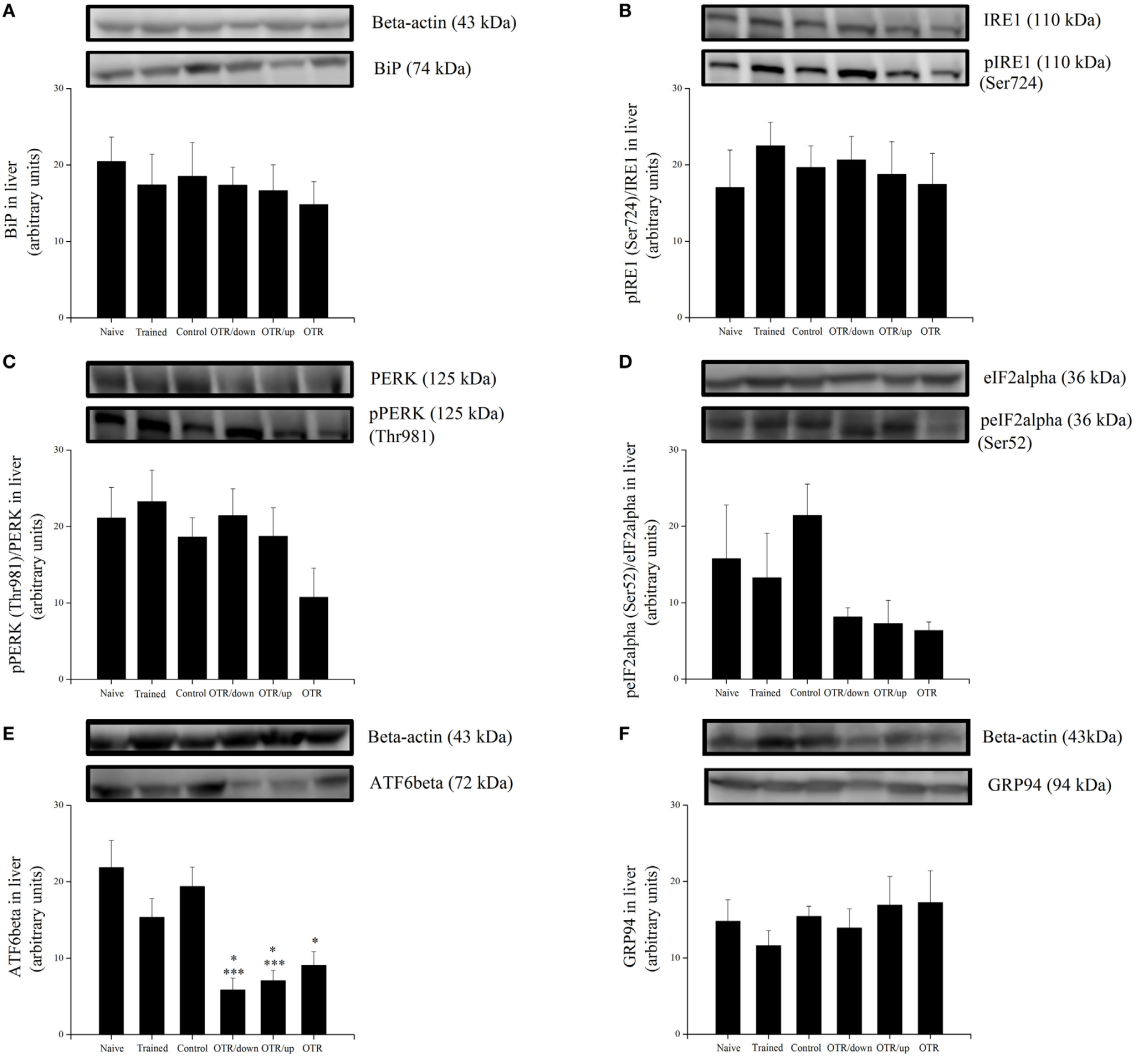
Protein levels (arbitrary units) of BiP **(A)**, pIRE1 (Ser724)/inositol-requiring enzyme 1 (IRE1) **(B)**, pPERK (Thr981)/protein kinase RNA-like endoplasmic reticulum kinase (PERK) **(C)**, peIF2alpha (Ser52)/eIF2alpha **(D)**, ATF6beta **(E)**, and glucose-regulated protein 94 (GRP94) **(F)** in the livers of experimental animals. Data are expressed as the mean ± SE of *n* = 6 mice. N, sedentary mice; TR, trained mice; CT, sedentary mice; OTR/down, overtrained by downhill running; OTR/up, overtrained by uphill running; OTR, overtrained by running without inclination. *P < 0.05 vs. CT group. ***P < 0.05 vs. N group. The original experiments are available in supplementary file 1 (i.e., BiP, peIF2alpha, eIF2alpha, and ATF6beta), file 2 (i.e., pIRE1, IRE1, Pperk, and PERK), and file 3 (i.e., GRP94).

**Figure 3:**
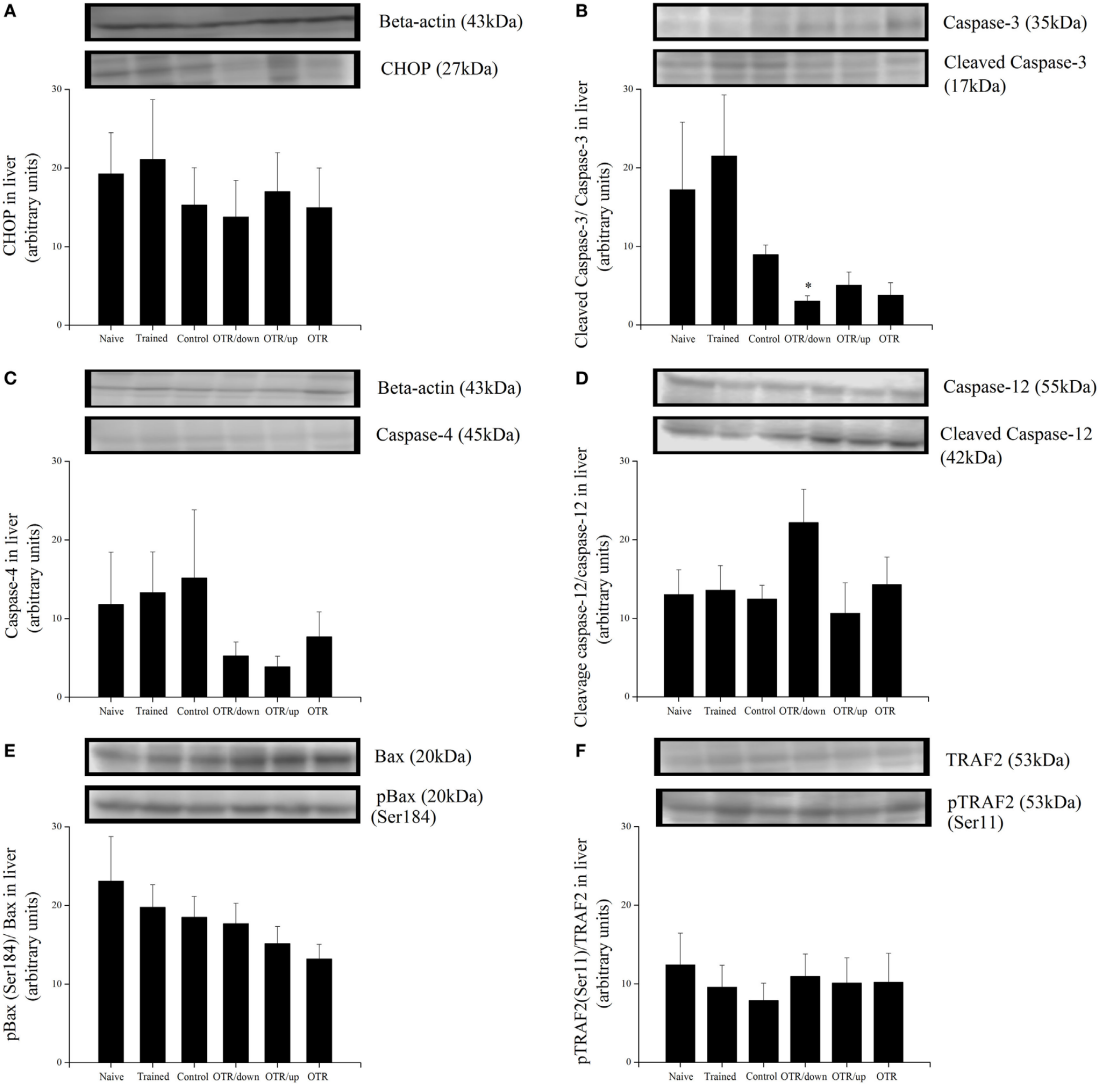
Protein levels (arbitrary units) of C/EBP-homologous protein (CHOP) **(A)**, Cleaved Caspase-3/Caspase-3 **(B)**, Caspase-4 **(C)**, Cleaved Caspase-12/Caspase-12 **(D)**, pBax (Ser184)/Bax **(E)**, and pTRAF2 (Ser11)/tumor necrosis factor receptor-associated factor 2 (TRAF2) **(F)** in the livers of experimental animals. Data are expressed as the mean ± SE of *n* = 6 mice. N, sedentary mice; TR, trained mice; CT, sedentary mice; OTR/down, overtrained by downhill running; OTR/up, overtrained by uphill running; OTR, overtrained by running without inclination. *P < 0.05 vs. CT group. The original experiments are available in supplementary file 3 (i.e., pBax and Bax), file 4 (i.e., Cleaved Caspase-12 and Caspase-12), file 5 (i.e., Cleaved Caspase-3 and Caspase-3), file 6 (i.e., Caspase-4), file 7 (i.e., pTRAF2), and file 8 (i.e., CHOP and TRAF2).

There was no significant difference between the experimental groups for the hepatic protein levels of BiP, pIRE1 (Ser724), pPERK (Thr981), peIF2alpha (Ser52), and GRP94 (Figures 2A–D,F, respectively). Figure 2E shows that the hepatic protein levels of ATF6beta were significantly lower (*P* < 0.05) in the OTR/down (73.2%), OTR/up (67.7%), and OTR (58.6%) groups compared to the N group. Also, the OTR/down and OTR/up groups were 69.8 and 63.6% lower (*P* < 0.05) compared to the CT group, respectively. There was no significant difference between the experimental groups for the hepatic protein levels of CHOP, caspase-4, cleaved caspase-12, pBax (Ser184), and pTRAF2 (Ser11) (Figures 3A,C–F, respectively). Figure 3B shows that the hepatic protein levels of cleaved caspase-3 were significantly lower (*P* < 0.05) in the OTR/down (66.0%) group compared to the CT group.

### Proteins Related to ER Stress and Apoptosis in the Heart

**Figure 4:**
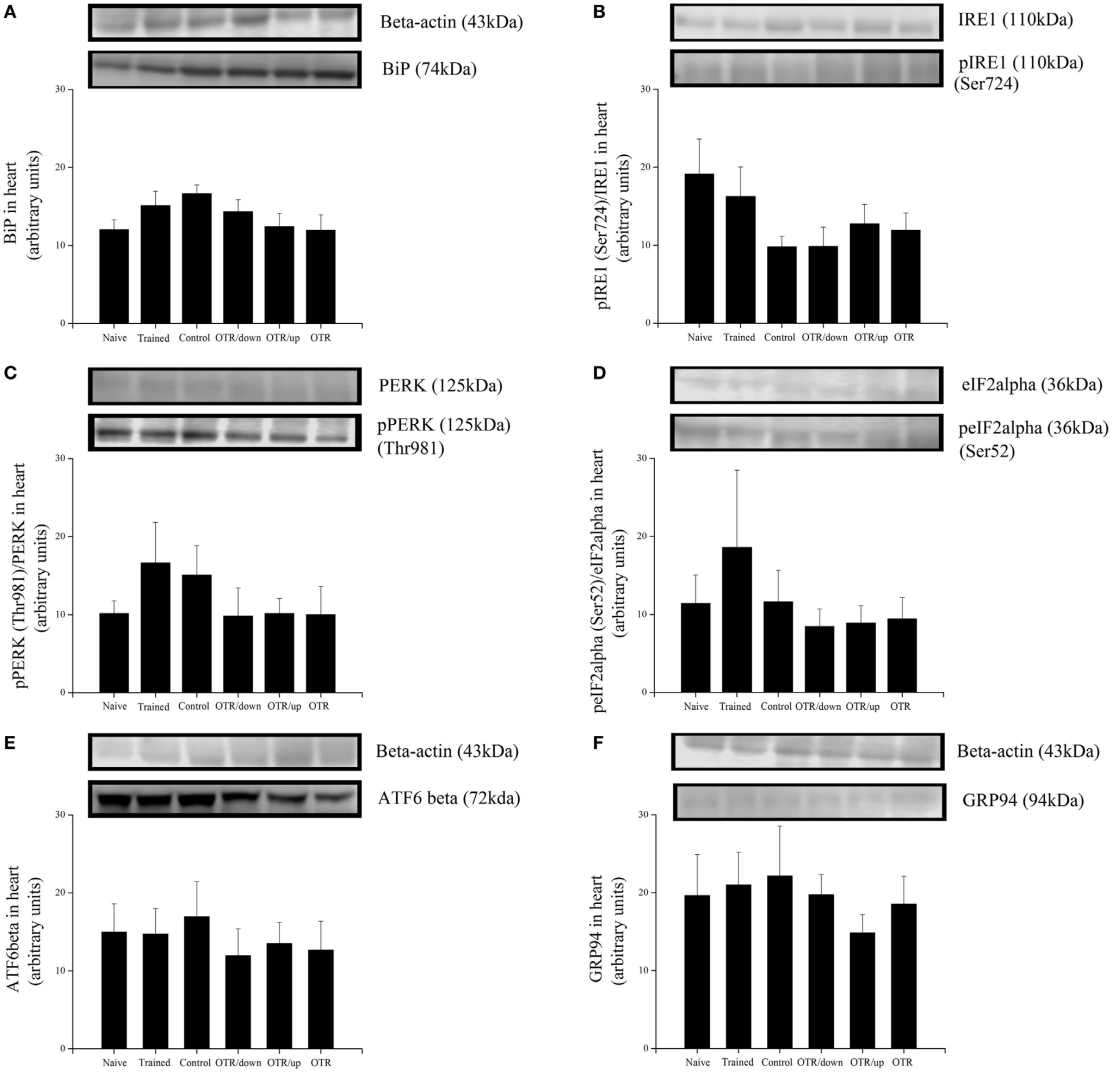
Protein levels (arbitrary units) of BiP **(A)**, pIRE1 (Ser724)/inositol-requiring enzyme 1 (IRE1) **(B)**, pPERK (Thr981)/protein kinase RNA-like endoplasmic reticulum kinase (PERK) **(C)**, peIF2alpha (Ser52)/eIF2alpha **(D)**, ATF6beta **(E)**, and glucose-regulated protein 94 (GRP94) **(F)** in the hearts of experimental animals. Data are expressed as the mean ± SE of *n* = 8 mice. N, sedentary mice; TR, trained mice; CT, sedentary mice; OTR/down, overtrained by downhill running; OTR/up, overtrained by uphill running; OTR, overtrained by running without inclination. The original experiments are available in supplementary file 9 (i.e., ATF6beta), file 10 (i.e., PERK), file 11 (i.e., Bip, pIRE1, and IRE1), files 12a.b and 12c.d (i.e., peIF2alpha, eIF2alpha and GRP94), and file 15 (i.e., pPERK).

**Figure 5:**
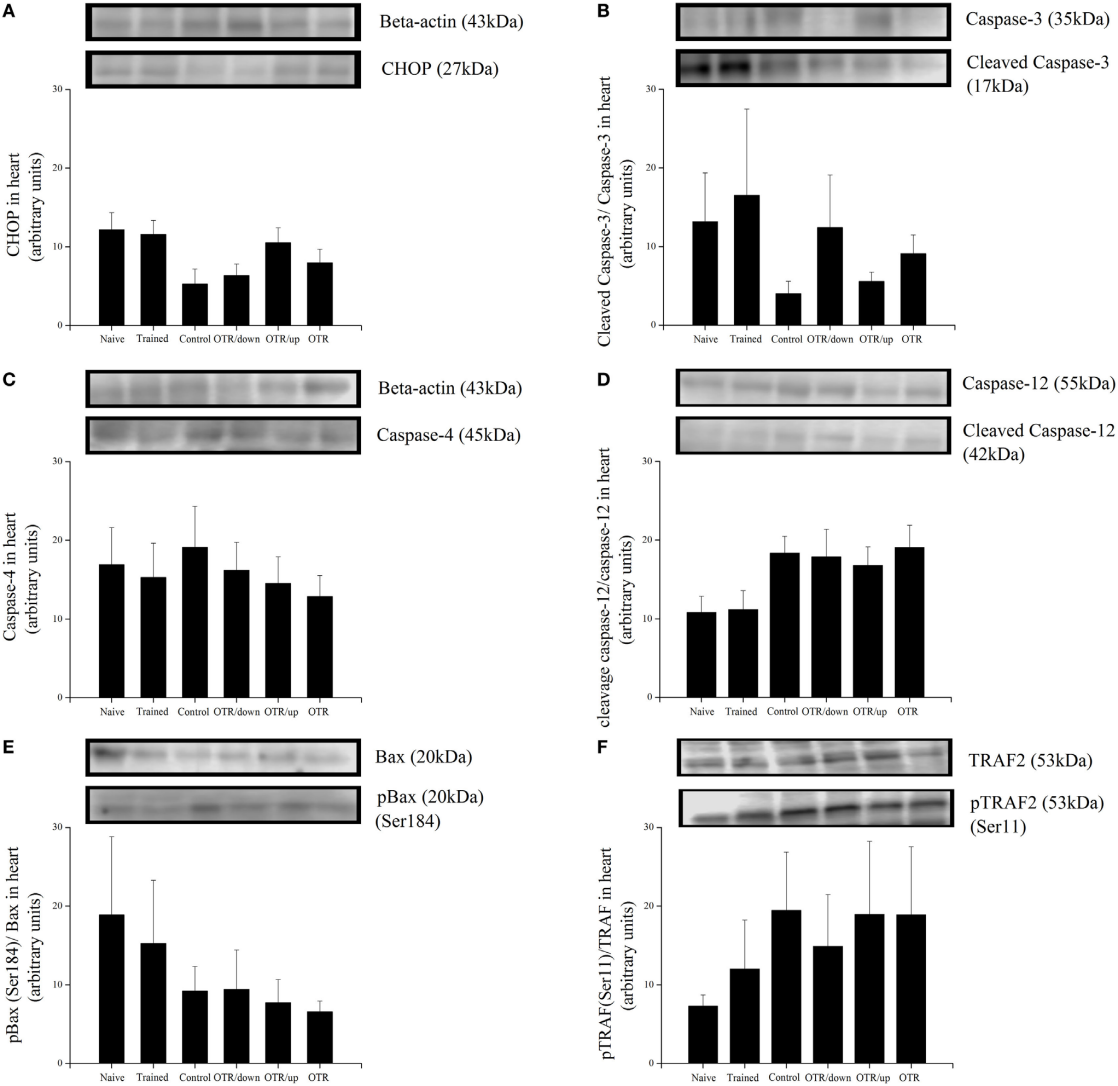
Protein levels (arbitrary units) of C/EBP-homologous protein (CHOP) **(A)**, Cleaved Caspase-3/Caspase-3 **(B)**, Caspase-4 **(C)**, Cleaved Caspase-12/Caspase-12 **(D)**, pBax (Ser184)/Bax **(E)**, and pTRAF2 (Ser11)/tumor necrosis factor receptor-associated factor 2 (TRAF2) **(F)** in the hearts of experimental animals. Data are expressed as the mean ± SE of *n* = 8 mice. N, sedentary mice; TR, trained mice; CT, sedentary mice; OTR/down, overtrained by downhill running; OTR/up, overtrained by uphill running; OTR, overtrained by running without inclination. The original experiments are available in supplementary file 10 (i.e., Caspase-4, Caspase-12 and Caspase-12), files 12a.b and 12c.d (i.e., Cleaved Caspase-3, Caspase-3, pBax and BAX), file 13 (i.e., TRAF2), file 14 (i.e., pTRAF2), and file 15 (i.e., CHOP).

There was no significant difference between the experimental groups for the cardiac protein levels of BiP, pIRE1 (Ser724), pPERK (Thr981), peIF2alpha (Ser52), ATF6beta, and GRP94 (Figures 4A–F, respectively). Also, the cardiac protein levels of CHOP, cleaved caspase-3, caspase-4, cleaved caspase-12, pBax (Ser184), and pTRAF2 (Ser11) were not significantly different between the experimental groups (Figures 5A–F).

## Discussion

This investigation tested the effects of different running OT protocols on hepatic and cardiac proteins related to ER stress and apoptosis in C57BL/6 mice. First, we observed that the OT models induced similar responses in rotarod, incremental load, exhaustive, and grip force tests. Second, the OT groups presented significantly lower hepatic ATF6beta values compared to the N group. Third, the OTR/down group displayed lower hepatic cleaved caspase-3 values compared to the CT group. Finally, the cardiac proteins related to ER stress and apoptosis were not modulated in response to the TR and OT protocols.

The TR and OT protocols presented the same characteristics during the first 4 weeks. Regarding rotarod and grip force performances from week 0 to week 4, the TR and OT models did not induce significant alterations compared to the CT group. From week 4 to week 8, while the TR protocol did not improve the rotarod and grip force performances, the OT models impaired these functional capacity parameters, as previously verified (33, 36, 37, 43). Using the same TR protocol, other researchers found no improvements in rotarod and grip force performances (44, 45). The rotarod test estimates cerebellar dysfunctions in rodents and may be influenced by cardiorespiratory endurance, motor coordination, and learning (46). As the TR group increased its ILT results, a parameter of cardiorespiratory endurance, from week 4 to week 8, it is possible that this particular protocol did not lead to significant alterations in motor coordination or learning.

In contrast, Huang and coworkers (47) observed that a running training protocol performed at 70% maximal oxygen uptake increased rotarod test values, which were associated with the upregulation of the dendritic density of Purkinje cells, a marker of motor coordination. The responses of the ILT and exhaustive test to the OT models from week 4 to week 8 are in accordance with other investigations performed by our research group (24, 33, 36, 37, 43) and reinforce the efficiency of these protocols to induce the NFOR state. Regarding the hepatic ER stress proteins, we found a downregulation of the ATF6beta protein levels in response to the OT protocols. ATF6 is an ER membrane-associated bZIP transcription factor, which restricts the transcriptional activity of sterol regulatory element-binding protein (SREBP) to decrease hepatic lipid stores (48).

Sterol regulatory element-binding protein-1c is the main transcription factor that regulates hepatic triglyceride synthesis, contributing to the pathogenesis of steatosis (49). Because ER stress may increase the transcription and mature form of SREBP-1c, this physiological phenomenon has been considered an activator of hepatic lipogenesis (50, 51). Usui and coworkers (52) found that diet-induced obese ATF6alpha-null mice displayed elevated mRNA expressions of SREBP1c, which was linked to a tendency for a higher degree of hepatic steatosis. Moreover, Yamamoto et al. (53) burdened ATF6alpha-knockout mice with an intraperitoneal injection of tunicamycin, an ER stress promoter, and observed hepatic dysfunction and steatosis. Zeng and coworkers (48) proposed a model in which glucose starvation induces the proteolytic cleavage of ATF6 that binds to SREBP2 in the nucleus and downregulates lipogenesis.

Recently, we found that the OTR/down and OTR/up models upregulated the hepatic SREBP-1 p125 precursor, but not the p68 mature form. Also, all of the OT protocols induced liver fat accumulation (25). Based on the previously described investigations (48, 52, 53), future studies should verify the effects of these running OT models on the hepatic levels of ATF6alpha and SREBP2, as well as on knockout models of ATF6alpha and beta, since these two isoforms play different roles in ER stress (54). The responses of hepatic ATF6 to exercise protocols are scarce (55, 56). For instance, Chapados and Lavoie (55) did not observe significant differences in ATF6 gene expression after 6 weeks of continuous running in female Sprague-Dawley rats. Recently, Tang and coworkers (56) verified that seven successive days of intense exercise increased the activity levels of ATF6 in mouse liver. The differences between the exercise protocols, experimental animals, and liver extraction times hamper the comparisons between the investigations above. However, even with the differences between the exercise protocols, we consider that the downregulation of ATF6 in the OT groups occurred due to the accumulation of hepatic fat, which was previously shown in the overtrained mice (48, 52, 53).

Regarding the hepatic apoptotic proteins, we found a downregulation of the cleaved caspase-3 after the OTR/down protocol. In contrast, several investigations did not observe significant alterations in cleaved caspase-3 hepatic levels after various acute and chronic exercise protocols (57–61). Only two studies examined the effects of exhaustive sessions on this apoptotic protein in the liver (57, 62). Mikami et al. (57) subjected sedentary and trained mice to an acute exhaustive running session and did not observe a significant change in the cleaved caspase-3 hepatic levels. Huang and coworkers (62) used an exhaustive treadmill exercise to study the effects of a medicinal mushroom on hepatoprotection, but they did not compare the cleaved caspase-3 content in the liver with a sedentary group.

Probably, the divergences between the studies reported above and the current investigation occurred due to the following reasons: (1) different exercise protocols—Mikami et al. (57) subjected their animals to a 4-week training period, while Huang et al. (62) subjected their animals to one exhaustive session after 30 days of *Ganoderma tsugae* supplementation. In the present study, the animals performed 8 weeks of OT; (2) experimental model of rodents—while Mikami et al. (57) and Huang et al. (62) investigated rats, we used C57BL/6 mice; (3) time for tissue extractions—Mikami et al. (57) used 48 h after the last exercise session, Huang et al. (62) euthanized the animals immediately after the exercise session, and we used 36 h after the grip force test. It is important to underline that the proteins and their targets are phosphorylated at different time periods (63).

Although the hepatic levels of cleaved caspase-12 for the OTR/down group were 70% higher compared to the N group, the differences between these groups were not significant. Recently, Ryoo (64) highlighted the importance of considering the timeline of events in which ER stress induced cell death. As the proteins and their targets are phosphorylated at different time periods (63), further studies should investigate the time-course of the hepatic proteins related to ER stress and apoptosis after the OT protocols to clarify whether they were activated before or after the current extraction time.

Based on the relationship between aerobic training, attenuated cardiac ER stress, and improved exercise capacity (14), we also verified whether the decrease in performance induced by our OT protocols was linked to ER stress and apoptosis in mice hearts. Herein, we found that the cardiac proteins related to ER stress and apoptosis were not modulated in response to the TR and OT protocols. Murlasits et al. (65) found that five exercise bouts performed at 70% maximal oxygen uptake for 60 min on consecutive days did not increase cardiac ER stress protein expressions in Sprague-Dawley rats. The authors emphasized that the cardiac extraction was performed 24 h after the last exercise session, and it was possible that the peak protein expression had occurred at a later point in time.

Recently, Bourdier and coworkers (16) verified that male Wistar rats subjected to 10 days of high-intensity training increased their cardiac levels of BiP without significant changes in pPERK, peIF2alpha, ATF4, ATF6, XBP1, CHOP, and cleaved caspase-3. The time interval between the last exercise session and cardiac muscle extraction performed by Bourdier et al. (16) was lower compared to the current investigation (i.e., 24 h versus 36 h). In fact, we selected 36 h of recovery after the grip force test to guarantee a minimum period to allow signaling pathways affected by exercise to recover to their basal levels. Also, our animals were submitted to a 12-h fasting period. Stressors like starvation cause BiP dissociation from the ER proteins (IRE1, PERK, and ATF6) (66). After release from BiP, the signal cascade is initiated for each of the ER proteins. Even after the fasting period, the proteins did not show significant changes.

Although moderate or high-intensity aerobic training can reduce cardiac ER stress induced by post-myocardial infarction heart failure or intermittent hypoxia (14, 16), respectively, their effects on healthy hearts are minimal. Modifications in redox status or reactive oxygen species (ROS) generation directly or indirectly affect ER homeostasis and protein folding. Under physiological conditions, ROS accumulation is protected by numerous endogenous antioxidant defense systems that include both enzymatic and non-enzymatic antioxidant mechanisms (67–69). One of the consequences of moderate exercise is to increase oxygen consumption, creating conditions for ROS generation and for oxidative stress in the organelle, cell, and tissue. Low physiological concentrations of ROS can regulate a variety of key mechanisms due to their roles as signaling molecules (70). Also, some tissues such as skeletal muscle have a well-developed system for regulating ROS, which includes mitochondrial and cytosolic isoforms such as superoxide dismutase (71). Recently, compared with the control mice, we showed that the animals subjected to the OTR/down protocol presented significantly higher levels of thiobarbituric acid reactive substance in the skeletal muscle cells. On the other hand, the glutathione levels (GSH) in the skeletal muscle cells were significantly lower in the OTR/down group than in the control and trained groups.

**Figure 6:**
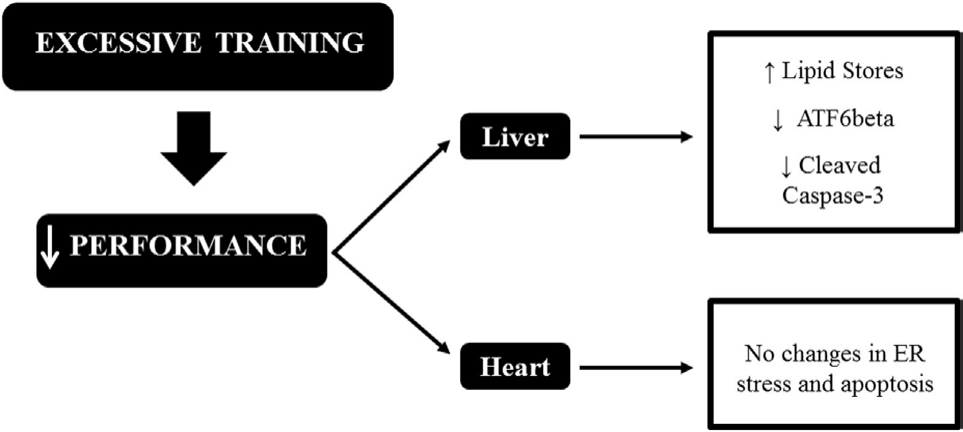
Schematic model summarizing the relationships between endoplasmic reticulum (ER) stress, apoptosis, and excessive training in the liver and heart of C57BL/6 mice.

To date, this is the first investigation regarding the effects of OT on the hepatic and cardiac proteins related to ER stress and apoptosis. In conclusion, the running OT protocols with the same external load, but performed downhill, uphill, and without inclination, decreased the levels of hepatic ATF6beta, which may be linked to high levels of liver fat, as recently published by our research group. Also, the OTR/down protocol decreased the levels of hepatic cleaved-caspase 3. Finally, the decrease in performance induced by our OT models is not linked to ER stress and apoptosis in mice hearts. These results are summarized in Figure 6.

## Ethics Statement

The experimental procedures were performed according to the Brazilian College of Animal Experimentation (COBEA) and were approved by the Ethics Committee of the University of Sao Paulo (ID 14.1.873.53.0).

## Author Contributions

Conceived and designed the experiments: DC, JP, LM, ER, and AS. Performed the experiments: AP, AR, LO, GM, and LV. Analyzed the data: AP and AS. Contributed reagents/materials/analysis tools: DC, JP, LM, ER, and AS. Wrote the paper: AP and AS.

## Conflict of Interest Statement

The authors declare that the research was conducted in the absence of any commercial or financial relationships that could be construed as a potential conflict of interest.
