# Supplementary material for: Levels of Hepatic Activating Transcription Factor 6 and Caspase-3 Are Downregulated in Mice after Excessive Training
Source: Front Endocrinol (Lausanne). 2017 Sep 26;8:247. doi: 10.3389/fendo.2017.00247 (PMC5622940; doi:10.3389/fendo.2017.00247)
Supplement: Supplementary file 8 [file image_8.pdf]

LIVER - Experiment 8

Membrane A

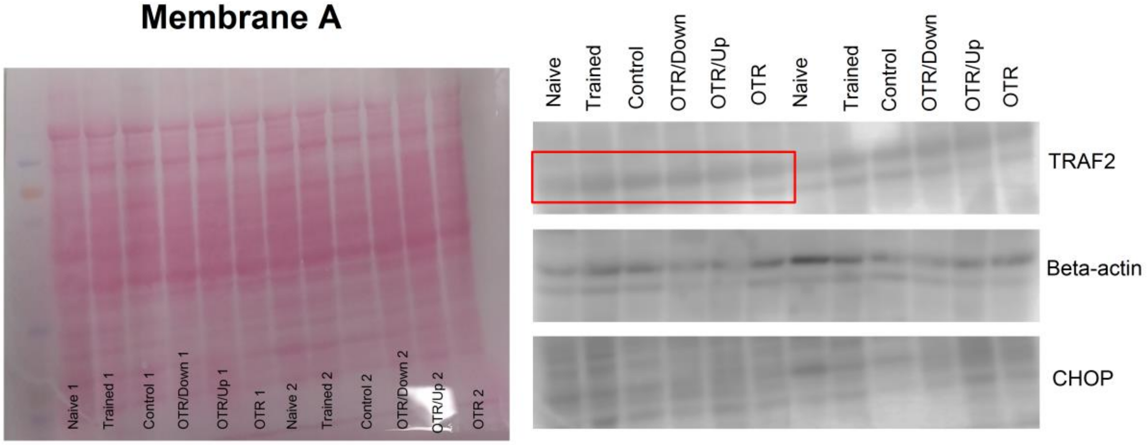

Membrane B

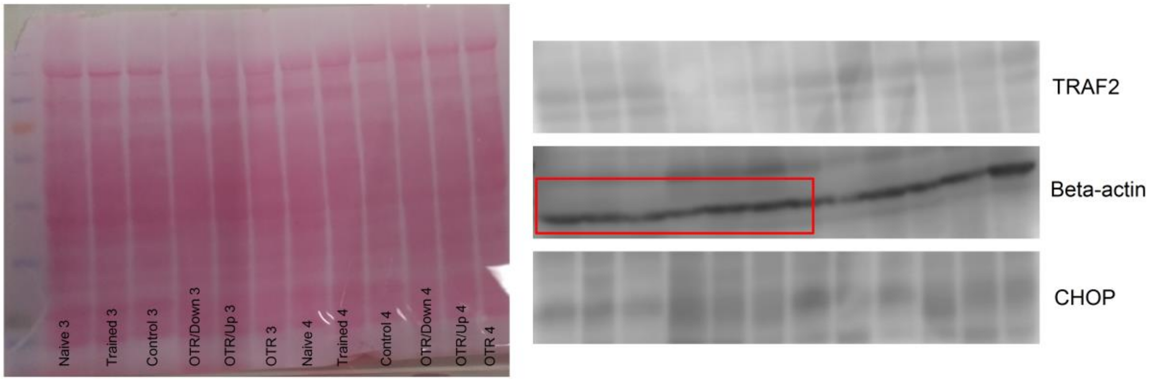

Membrane C

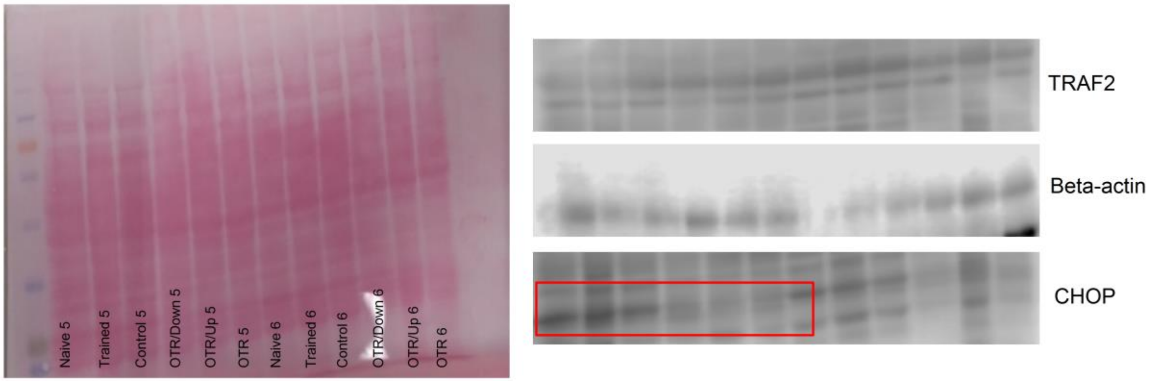

Supplementary file 8. Original experiments performed for TRAF2, Beta-actin and CHOP. The bands outlined in red were used as representative in the manuscript figures. The run and transfer process of membranes A, B and C were performed at the same time.
