# Supplementary material for: Levels of Hepatic Activating Transcription Factor 6 and Caspase-3 Are Downregulated in Mice after Excessive Training
Source: Front Endocrinol (Lausanne). 2017 Sep 26;8:247. doi: 10.3389/fendo.2017.00247 (PMC5622940; doi:10.3389/fendo.2017.00247)
Supplement: Supplementary file 11 [file image_11.pdf]

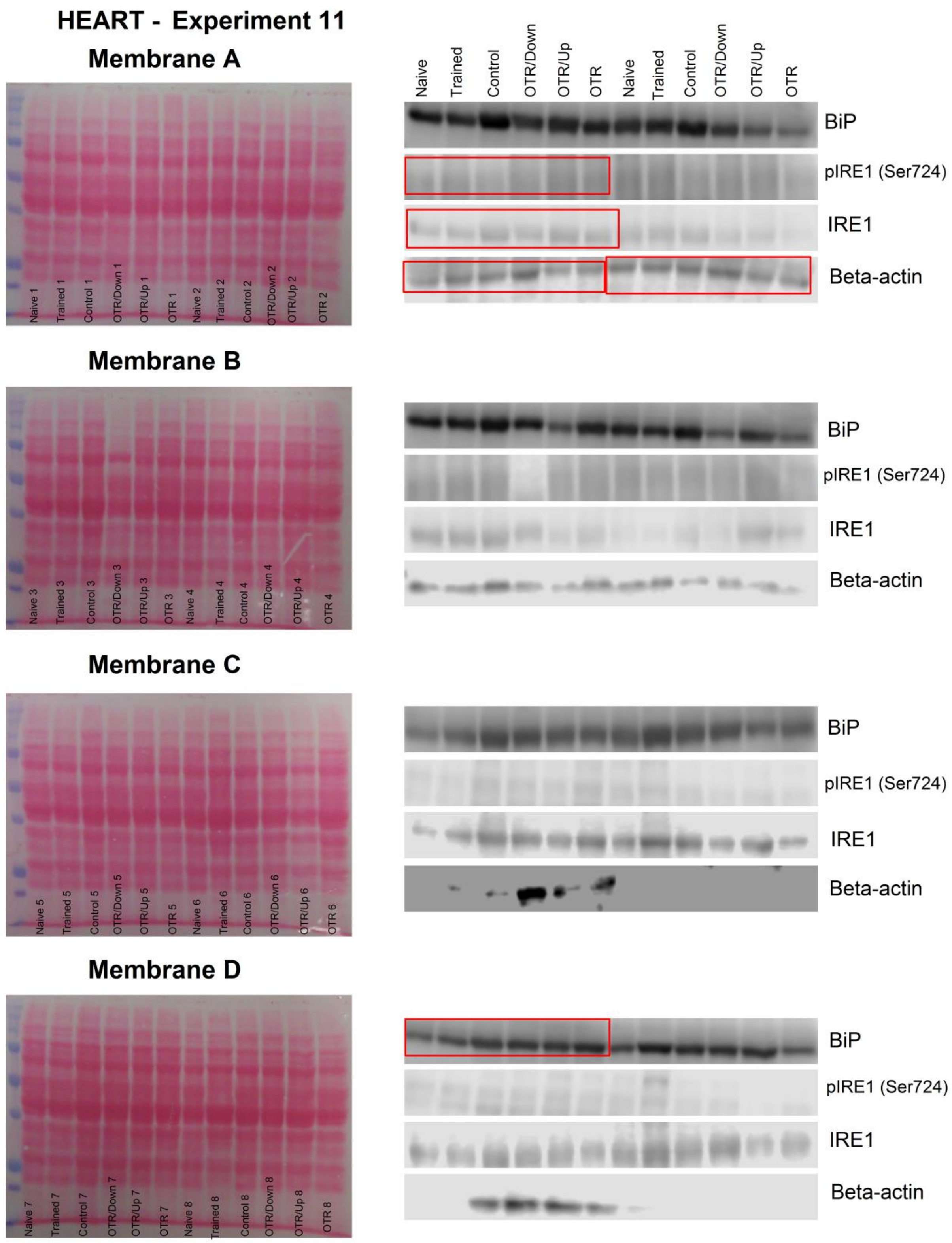

Supplementary file 11. Original experiments performed for BiP, pIRE1 (Ser724), IRE1 and Beta-Actin. The bands outlined in red were used as representative in the manuscript figures. The run and transfer processes of membranes A, B, C and D were performed at the same time.
